# Correlation between Speech Perception Outcomes after Cochlear Implantation and Postoperative Acoustic and Electric Hearing Thresholds

**DOI:** 10.3390/jcm10020324

**Published:** 2021-01-17

**Authors:** Ursina Rüegg, Adrian Dalbert, Dorothe Veraguth, Christof Röösli, Alexander Huber, Flurin Pfiffner

**Affiliations:** 1 Department of Otorhinolaryngology, Head and Neck Surgery, University Hospital Zurich, 8091 Zurich, Switzerland; ursina.rueegg@usz.ch (U.R.); adrian.dalbert@usz.ch (A.D.); dorothe.veraguth@usz.ch (D.V.); christof.roeoesli@usz.ch (C.R.); alex.huber@usz.ch (A.H.); 2 Department of Otorhinolaryngology, University of Zurich, 8091 Zurich, Switzerland

**Keywords:** speech perception, hearing test, cochlear implant, audiometry, electrocochleography

## Abstract

The reliable prediction of cochlear implant (CI) speech perception outcomes is highly relevant and can facilitate the monitoring of postoperative hearing performance. To date, multiple audiometric, demographic, and surgical variables have shown some degree of correlation with CI speech perception outcomes. In the present study, postsurgical acoustic and electric hearing thresholds that are routinely assessed in clinical practice were compared to CI speech perception outcomes in order to reveal possible markers of postoperative cochlear health. A total of 237 CI recipients were included in this retrospective monocentric study. An analysis of the correlation of postoperative pure-tone averages (PTAs) and electric CI fitting thresholds (T-/C-levels) with speech perception scores for monosyllabic words in quiet was performed. Additionally, a correlation analysis was performed for postoperative acoustic thresholds in intracochlear electrocochleography (EcochG) and speech recognition scores in a smaller group (*n* = 14). The results show that neither postoperative acoustic hearing thresholds nor electric thresholds correlate with postoperative speech perception outcomes, and they do not serve as independent predictors of speech perception outcomes. By contrast, the postoperative intracochlear total EcochG response was significantly correlated with speech perception. Since the EcochG recordings were only performed in a small population, a large study is required to clarify the usefulness of this promising predictive parameter.

## 1. Introduction

Multiple audiometric, demographic, and surgical variables have shown some degree of correlation with cochlear implant (CI) speech perception outcomes. The duration of deafness, etiology of hearing loss, and preoperative speech understanding are known to influence postoperative speech perception outcomes [1,2,3,4,5,6]. However, the results for cognitive factors [7,8] and age at implantation in adult CI users [1,2,9,10] have been inconclusive. Nevertheless, even the above-mentioned variables show only limited abilities to explain or predict large variabilities in CI speech recognition outcomes. A reliable outcome measure is thus highly relevant and would allow the monitoring of postoperative hearing performance to identify CI users showing deviations from the expected outcome and lead to the adjustment of postoperative follow-up therapy.

To enhance speech recognition with a CI system, the presence of functional neural fibers is crucial. The electrical stimulation of neural populations depends on the presence of preserved neural and sensory structures (cochlear health) after cochlear implantation and the efficiency of the stimulation of these structures [11,12,13]. The hypothesized and investigated markers that reflect postoperative cochlear health and neural stimulability include (1) the degree of preserved residual acoustic hearing, (2) CI fitting parameters such as the electric hearing threshold levels and electric dynamic range (eDR), and (3) the level of postoperative acoustically evoked potentials measured by electrocochleography (ECochG).

The results of studies investigating the correlation between preserved residual acoustic hearing and speech perception in electric-only auditory stimulation are conflicting [14,15,16,17]. While Carlson et al. [15] and Dalbert et al. [16] noted an improved understanding of speech if residual acoustic hearing was preserved, Balkany et al. [14] and Cosetti et al. [17] could not demonstrate this effect. To date, most reports have mainly compared hearing preservation, which depends on the preoperative acoustic hearing, to postoperative speech outcomes, and not postoperative acoustic thresholds. In this study, we investigated whether postoperative hearing thresholds can serve as a better marker of cochlear health that correlates with speech outcomes.

The correlation between electric hearing threshold levels and speech outcomes is not clear. De Graaff et al. [18] and Van der Beek et al. [19] noted a possible correlation with speech outcomes, in contrast to other studies [20,21]. In particular, studies with large numbers of participants have not included postoperative markers of cochlear health such as acoustic and electric hearing thresholds [1,2].

Moreover, the results for intraoperative acoustically evoked potentials measured by electrocochleography (ECochG) before electrode array insertion have been reported to correlate with speech perception outcomes [11,22,23]. To date, correlations between postoperative ECochG and speech outcomes have not been reported and are a possible mode for future routine clinical measurements driven by recent advancements in CI systems [24,25].

In this retrospective study, we aimed to investigate the correlation between speech recognition outcomes and markers of cochlear health that were assessed in routine clinical evaluations. The investigated markers were postoperative acoustic hearing thresholds and electric fitting thresholds from regular CI fitting assessments in a large population. In a smaller group of patients, the correlation analysis of postoperative intracochlear ECochG responses and speech recognition scores was performed in order to evaluate the usefulness of ECochG recordings in future clinical routine monitoring for CI outcomes.

## 2. Experimental Section

### 2.1. Study Participants

The participants included in this retrospective study underwent implantation between 2004 and 2017 at the cochlear implant center in Zürich, Switzerland. The Ethics Committee of the Canton of Zurich approved the study protocol (KEK-ZH No. 2015-0430) in accordance with the Helsinki Declaration.

To qualify for inclusion, participants in group I were required to have undergone implantation with a CI system of generation CIC4 (Nucleus Implant System, Sydney, Australia), completed postoperative pure-tone audiography (PTA), and completed the Freiburg monosyllabic test between 1 and 5 years after implantation. Patients using fitting programs (maps) with more than eight inactive electrode contacts or with pulse widths larger than 75 µs were excluded. The inclusion criteria for group II were residual hearing at low frequencies, available postoperative ECochG recording data, implantation with a HiRes™ Ultra (Advanced Bionics LLC, Valencia CA, USA), and a completed postoperative Freiburg monosyllabic test.

For patients with bilateral implants, both ears were analyzed separately, and in the re-implanted patients, only data for the most recent implant were included. The size of the population (group 1, *n* = 237; group 2, *n* = 14) refers to the number of implanted ears. Table 1 summarizes the demographic data for each group.

### 2.2. Audiometric Evaluation

Postoperative pure-tone audiometry was performed in accordance with ISO standard 8253-1. Acoustic thresholds were measured at 125 Hz, 250 Hz, 500 Hz, 1 kHz, 2 kHz, 4 kHz, 6 kHz, and 8 kHz; the corresponding maximum audiometer output for each frequency was 85, 100, 120, 120, 120, 120, 115, and 105 dB HL, respectively. Vibrotactile feedback from the participants was considered to indicate no response. If no hearing was detectable, 5 dB was added to the maximum audiometer output for the calculation of the average hearing threshold. The postoperative pure-tone average (pPTA) was defined as the average of all the measured frequencies. The mean assessment point was 7 months (SD, 15 months) after implantation.

Aided speech understanding in quiet was measured using monosyllabic words from the Swiss version of the German Freiburg test [26]. Word recognition scores at 65 dB sound pressure level (SPL) from 12 months or more after implantation were determined from the speech signal presented from the front. The mean assessment point in time was 21 months (SD 11) for group I and 15 months (SD 2) for group II.

### 2.3. Electric CI Hearing Thresholds

Electric CI fitting thresholds were measured by experienced audiologists during routine follow-up assessments according to the procedures recommended by the manufacturer. Hearing thresholds (T-levels) and comfortable loudness thresholds (C-levels) in current level (CL) units from the clinical fitting software (Cochlear Custom Sound Suite, version 4.4, Cochlear Ltd., Sydney, NSW, Australia) of the most recent fittings were analyzed. Only fittings that had been implanted for at least 12 months were analyzed, since by 12 months, patients typically reach a point where only minor CI fitting adjustments are required. 

CL units are defined as the amount of electrical current delivered to the implant expressed in clinical programming units from 0255 and dependent on the pulse width (PW) and stimulation rate. To allow comparisons between individual patients, T- and C-level values were converted for a PW of 25 µm according to the formula:

(1) $$\mathrm{Current}\;I\left[\mu A\right]=\;17.5\times 100^{\frac{\mathrm{level}\left[\mathrm{CL}\right]}{255}}\;;\;\mathrm{level}[\mathrm{CL}]\;=\;T-/C-\mathrm{level}\;\mathrm{setting}\;\mathrm{in}\;\mathrm{fitting}\;\mathrm{software}$$

(2) $$\mathrm{Current}\;I_{cor}\left[\mu A\right]=I\frac{PW_{ref}}{PW}PW_{ref}=25\;\mu \mathrm{ms};\;\mathrm{PW}\;=\;\mathrm{pulse}\;\mathrm{width}\;\mathrm{setting}\;\mathrm{in}\;\mathrm{fitting}\;\mathrm{software}$$

whereby the current Icor was transformed back into a T-/C-level in CL units with a reference PW of 25 µs.

The T- and C-level values were converted for a stimulation rate of 900 pulses per second (pps) in accordance with the manufacturer’s recommendations and references [27,28]. The mean T- and C-level values were defined as the average thresholds over all the active electrode contacts. The mean electric dynamic range (eDR) was defined as the difference between the corrected mean T- and C-levels. 

For the correlation between the speech recognition scores and C-level, the C-level was additionally corrected according to the individual volume setting during the speech understanding test according to the manufacturer’s specifications:

(3) $$Clevel_{vol\_cor}=Tlevel+eDR\times Volume_{cor}$$

(4) $$Volume_{cor}=0.8+0.2\frac{Vol}{10}\;;\;\mathrm{Vol}\;=\;\mathrm{volume}\;\mathrm{setting}\;\mathrm{in}\;\mathrm{fitting}\;\mathrm{software}\;(\mathrm{number}\;\mathrm{between}\;1\;\mathrm{and}\;10)$$

### 2.4. Electrocochleography

Postoperative intracochlear EcochG signals (group II) were recorded in accordance with a previously described method [29]. The most apical electrode contact was used to measure intracochlear EcochG signals through the cochlear implant system (Bionic Ear Data Collection System, version 1.18; Advanced Bionics, Valencia, CA, USA) using the Clarion Programming Interface (Advanced Bionics) and a Platinum Series Speech Processor. Considering the predominance of low-frequency hearing, EcochG signals at low frequencies (250, 500, and 1000 Hz tone-burst signals) were recorded at the maximum acceptable sound pressure levels or maximal 110 dB SPL. Averaged responses for rarefaction and condensation stimulus phases were stored separately. Difference curves (the subtraction of the condensation phase from the rarefaction phase) and alternating curves (the sum of both phases) were calculated. On the basis of a previous study [23], the frequency spectrum amplitudes of the difference and sum curves were analyzed; i.e., the sum of the spectrum amplitude of the stimulus signal and its first harmonic was defined as the ongoing EcochG response. The sum of the magnitudes of ongoing EcochG responses at all three frequencies (250, 500, and 1000 Hz) was termed the total EcochG response.

### 2.5. Statistical Analysis

Correlation analyses and illustration were performed using GraphPad Prism (Version 8.0.0; GraphPad Software Inc., San Diego CA, USA). The data were analyzed for each of the two study groups separately. The used statistical test (Spearman) shows the correlation between the rankings of the two investigated variables to assesses how well the relationship can be described using a monotonic function.

## 3. Results

### 3.1. Audiometric Evaluation

**Figure 1:**
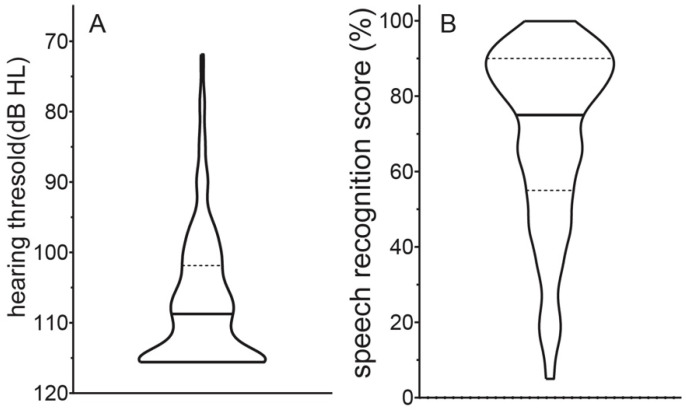
Distribution of postoperative pure-tone averages ((**A**), left) and speech recognition scores ((**B**), right) represented as a violin plot with the medians (horizontal plane lines) and interquartile ranges (horizontal dotted lines).

The mean pPTA was 106.9 dB HL (SD, 9.3; range, 71.8–115.6 dB HL; Figure 1A). For 51 ears (21%), a pPTA better than 100 dB HL was measured, and no measurable hearing thresholds at any frequency were seen in 68 (28.7%) ears. The mean postoperative word recognition score for monosyllabic words in quiet was 69.7% (SD, 23.6; range, 5–100%; Figure 1B).

### 3.2. Electric CI Hearing Thresholds and Electric Dynamic Range

**Figure 2:**
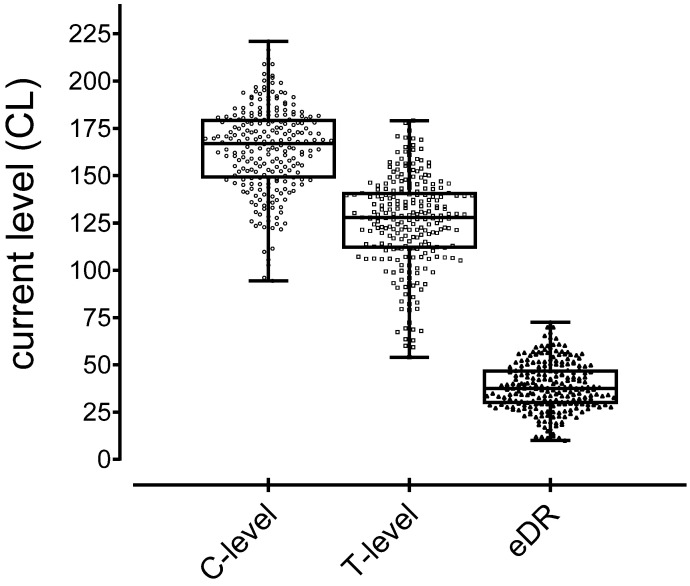
Scatter boxplot with whiskers from the minimum to maximum C-level (**left**), T-level (**middle**), and electric dynamic range (**right**).

Figure 2 shows the T-levels (mean, 125.6 CL; SD, 24; range, 54–179) and C-levels (mean, 164.1 CL; SD, 23; range, 94–221) with the resulting eDRs (mean, 38.5 CL; SD, 13; range, 10–73). 

### 3.3. ECochG Responses and Speech Understanding

The mean postoperative word recognition score was 49.34% (SD, 25.4%; range, 0–85%) for group II. The mean total ECochG response was 13.3 µV (SD, 17.7 µV; range, 0–55 µV). One of the 14 participants included in the statistical analysis showed no ECochG response despite showing measurable postoperative hearing.

### 3.4. Correlations

**Figure 3:**
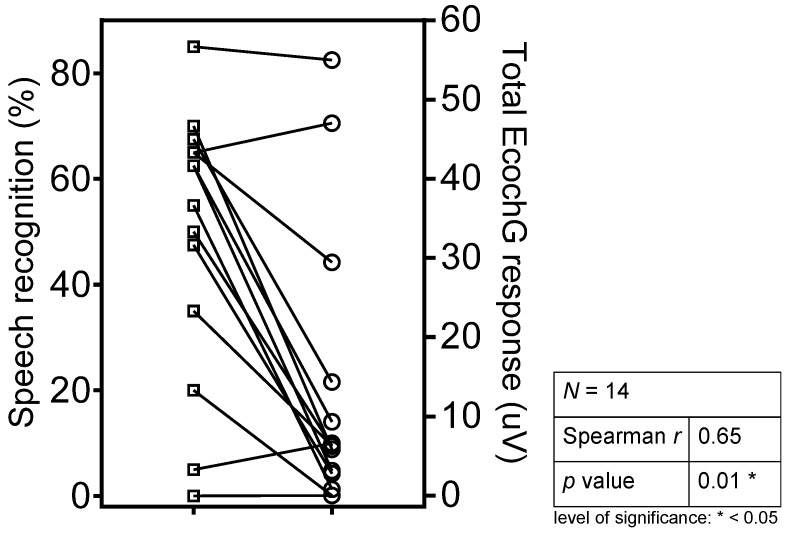
Spearman’s rank correlation between total EcochG response recordings and speech recognition.

The pPTA (Spearman *r* = 0.06, *p* = 0.32), T-level (Spearman *r* = 0.09, *p* = 0.16), and eDR (Spearman *r* = 0.10, *p* = 0.14) did not correlate with the speech recognition scores (Table 2). There was a significant correlation between the speech outcome scores and C-levels (*p* = 0.02*) with a low Spearman value (*r* = 0.15), indicating that the variability in the outcome score is only weakly predicted. A strong correlation was observed between the total ECochG response and speech recognition scores (Spearman *r* = 0.65, *p* = 0.01, Figure 3).

## 4. Discussion and Conclusions

The aim of the present study was to investigate routinely assessed postsurgical parameters to evaluate their suitability for monitoring CI outcomes. Additionally, a potential routine postoperative parameter, the ongoing ECochG response, and its correlation with CI speech perception scores was investigated. Although the postoperative acoustic hearing thresholds and electric fitting parameters, including the T-level, eCT, and eDR, did not allow a prediction of word perception, the postoperative total ECochG response was significantly correlated with the speech perception scores.

Our result with a large population (*n* = 237) confirms the findings of other research groups [18,19,20] that demonstrated no significant effects of acoustic hearing on speech perception with smaller sampling sizes. Cosetti et al. [17] noted that word perception was correlated neither with preserved residual hearing nor with postoperative acoustic hearing thresholds. Their findings support the results obtained by D’Elia et al. [21], who found no significant difference in CI performance between patients with good (90 dB or less) or poor (above 90 dB) preoperative acoustic low-frequency hearing thresholds. Likewise, Balkany et al. [14] showed no significant correlation of the hearing preservation after implantation with the understanding of speech in a group of 28 patients. Our findings, however, stand in contrast to those reported by Carlson et al. [15], who observed a significant correlation between hearing preservation and speech perception, although one inclusion criterion in their study was a preoperative low-frequency hearing threshold maximum of 70 dB HL at 250 Hz. Residual hearing additional to pure electric CI hearing can lead to better performance in speech testing and may increase the correlation with hearing preservation. By contrast, in the present study, we intentionally included patients regardless of their hearing thresholds. In fact, over 80% of the measured ears in our study suffered from hearing loss, with an air conduction threshold above 100 dB HL. Presumably, in these patients, the extent of surviving structures was minimal. As a result, the inner ear may not have reacted sensitively to minimal changes in electric stimulation. Similarly, the study by Dalbert et al. [16] demonstrated a correlation between preserved acoustic hearing and speech perception after cochlear implantation. Their group calculated the hearing preservation taking into account the pre- and postoperative acoustic hearing, which they then correlated with speech perception. On the other hand, in the present study, we correlated pPTA directly with speech perception to illustrate its possible use as a prediction parameter.

The analyzed values of the T-/C-levels measured in the present study (mean T-level = 126 CL and C-level = 164 CL) are comparable to the findings reported previously [18] (18), with T-levels of 120 CL (late onset) and 127 CL (in the early onset) and C-levels of 171 and 172 CL for early and late onset, respectively. D’Elia et al. [21] and Kim et al. [20] reported higher mean T/C-levels (T-level = 140 CL [21]; T-levels of 142 to 144 CL in prelingual and postlingual patients, respectively, and C-levels up to 183 [20]). Blamey et al. [30] reported lower T-levels (117.4 CL for middle electrodes). These differences can be primarily attributed to the fact that not all groups underwent correction of T-/C-levels for rate and PW and that these studies used different CI fitting procedures to determine T/C hearing thresholds.

Consistent with our findings, electric parameters (T-/C-levels and eDR) do not explain much of the variability in the outcome scores as reported with smaller population groups [21,31,32]. The eDR was not significantly different between groups of patients with good (from 80%) and worse (below 79%) open-set speech perception in quiet [31]. Accordingly, D’Elia et al. could not demonstrate a significant correlation of a wider eDR with an improved understanding of speech in an open setting [21]. Other research groups revealed only weak associations or associations restricted to specific circumstances [19,20,33,34]. An increase in T-levels, leading to smaller eDRs, correlated inversely but only slightly with consonant and vowel perception in a study investigating the consequences of errors in the CI setting [34]. A change of 30% in the eDR did not affect speech, and if the eDR was expanded by 60% or more, even a decrease in speech perception in quiet was displayed, presumably as a result of lower T-levels [33]. In long-term CI users (≥5 years), the eDR was only associated with phonetically balanced words and consonant perception but not with sentence and vowel perception. Furthermore, the T-level was not correlated at all with any of the speech measurements [20].

In contrast to our results, Loizou et al. [35] reported that a wide eDR increases the understanding of speech, due to the precise transformation of sound levels into electric impulses that allow for the differentiated understanding of speech [30,36]. Consistent with these findings, another study [18] demonstrated the positive influence of a large eDR (>40 CL) on speech perception in patients with late-onset hearing loss, as well as that of a low T-level (<120 CL) on speech perception in patients with early-onset severe hearing impairment. However, there are still several differences between our study and the study by de Graaff et al. First, we did not split our patients based on their onset of hearing impairment, and their study population was approximately half of our study population. Furthermore, the fitting procedures differ among CI centers, and the results are thus not unconditionally comparable.

The postoperative intracochlear ECochG recordings showed a good correlation with the speech recognition scores. A previous study [23] analyzed extracochlear ECochG recordings just before cochlear implantation in a large population (*n* = 97), and obtained a strong correlation (*r* = 0.682, *p* < 0.001) after comparisons with speech perception scores 6 months after implantation. Consistent with those results [23], we found a strong correlation (*r* = 0.65, *p* = 0.01) between postoperative ECochG recordings and speech perception, although our findings were preliminary in nature and obtained in a smaller population (*n* = 14). The measurement methods in the two studies differed as well; in comparison with the extracochlear recordings obtained before implant insertion in the previous study, the use of postoperative intracochlear measurements in our study offered several advantages. Primarily, the recordings were obtained closer in time to speech testing and allowed for more precise comparisons considering the possible changes in inner ear structures during CI insertion. Second, intracochlear recordings are closer to the source of the generator and may more precisely represent the remaining inner ear structures. As a result, the correlation with speech perception of intracochlear signals after implantation is expected to be a stronger marker of cochlear health. Moreover, using the implanted electrode as a recording electrode allows a noninvasive, short, and simple technique that can therefore be used for routine clinical assessment. In that sense, postoperative ECochG recording may represent an additional postoperative assessment for monitoring the performance of CI recipients in the future.

However, the clinical relevance of EcochG data for CI users has been reported in the literature according to a recent systematic review [37], which shows hearing preservation as an important and highly discussed topic. Correlation analyses between postoperative hearing thresholds and EcochG recordings have been reported in the literature [38,39,40] and were not part of this study.

As a limitation of the present study, the method for measuring speech outcomes must be mentioned. Residual acoustic hearing adds to electric-only hearing and can lead to better speech understanding scores. However, in the present study, only six subjects (2.5% of the population) had a pPTA better than 80dB HL, and hence, the influence on the presented correlation analyses was very low.

Monosyllabic speech testing in quiet does not reflect the understanding of speech in daily life. However, our aim in this study was to find a correlation between available data collected routinely in the clinic in a large number of CI clinics rather than speech tests in more complex acoustic environments. Furthermore, the benefits of postoperative acoustic hearing are not limited to improved speech recognition. It has been reported that residual hearing leads to an overall increase in quality of life in various ways such as self-confidence, safety, and sound awareness when the CI is switched off; improved music perception; increased feelings of safety; and well-being.

The outcome of the present study suggests that postoperative acoustic and electric thresholds do not serve as reliable markers for CI outcomes. As with the other predictive parameters reported in the literature, the influence of these postoperative acoustic and electric hearing thresholds as prediction factors for speech outcomes alone is not sufficiently reliable for use for monitoring the speech recognition scores of CI users. The postoperative intracochlear EcochG recordings showed a clear trend toward a positive correlation with speech perception outcomes in the CI-only condition after cochlear implantation. Since EcochG recordings were only performed in a small population, there is a need for a large study to clarify this promising predictive parameter.

## Figures and Tables

**Table 1:** Participants’ demographic data.

	Group I	Group II
Total (*n*)	237	14
Age at implantation (years: mean, (SD))	47 (21)	56 (16)
Sex (% female)	57	36
Implant side (% right)	52	57
Bilateral implanted participants (*n*)	25	0
Cochlear implant model		
Cochlear™ Nucleus^®^ 512	71	
Cochlear™ Nucleus^®^ 422	49	
Cochlear™ Nucleus^®^ 522	17	
Cochlear™ Nucleus^®^ 24RE(CA)	100	
Advanced Bionics HiRes 90KTM Advantage		11
Advanced Bionics HiResTM Ultra		3

**Table 2:** Spearman’s correlation of postoperative pure-tone average (pPTA), T-level, C-level, and electric dynamic range eDR with speech recognition.

	Speech Recognition (%) vs. pPTA (dB HL)	Speech Recognition (%) vs. T-Level (CL)	Speech Recognition (%) vs. C-Level (CL)	Speech Recognition (%) vs. eDR (nC)
Spearman *r*	−0.06	0.09	0.15	0.10
*p*-value	0.32	0.16	0.02 *	0.14

Level of significance: * < 0.05.
